# To Quiz or to Shoot When Practicing Grammar? Catching and Holding the Interest of Child Learners: A Field Study

**DOI:** 10.3389/fpsyg.2022.856623

**Published:** 2022-04-14

**Authors:** Cyril Brom, Lukáš Kolek, Jiří Lukavský, Filip Děchtěrenko, Kristina Volná

**Affiliations:** ^1^Faculty of Mathematics and Physics, Charles University, Prague, Czechia; ^2^Faculty of Arts, Charles University, Prague, Czechia; ^3^Institute of Psychology, Czech Academy of Sciences, Prague, Czechia; ^4^Development and New Media Department (decko.cz and ctart.cz), Czech TV, Prague, Czechia

**Keywords:** game-based learning (GBL), quiz, intrinsic integration, learning analytics, persistence, interest, distraction

## Abstract

Learning grammar requires practice and practicing grammar can be boring. We examined whether an instructional game with intrinsically integrated game mechanics promotes this practice: compared to rote learning through a quiz. We did so “in the field.” Tens of thousands children visited, in their leisure time, a public website with tens of attractive online games for children during a 6-week-long period. Of these children, 11,949 picked voluntarily our grammar training intervention. Thereafter, unbeknown to them, they were assigned either to the game or the quiz condition. By means of learning analytics, we examined variables related to participants’ persistence and performance. The results showed large participant drop-out before completing the first level in both conditions (42.2%), confirming the boringness of the topic. More children completed at least one level in the game compared to the quiz (61.8 vs. 53.6%). However, more children completed the intervention (all six levels) with the quiz (6.0 vs. 4.3%). In the game, children answered fewer questions correctly (36.3 vs. 47.4) and made more errors compared to the quiz (16.1 vs. 13.1). These findings suggest that even if a game initially catches user attention, it may not hold it. Plus, even if it is a minimalistic game with intrinsic integration of learning and playing, it may be distractive. We conclude that persistence in practicing grammar may be driven by other means than by a game’s shooting mechanics; for instance, by a desire to learn the topic and a feeling of achievement or by quizzing mechanics.

## Introduction

Harnessing motivational power of games to enhance learning is a long-standing goal of educational game designers (cf. Malone, 1981; Cordova and Lepper, 1996; Garris et al., 2002). Motivation has many facets (e.g., Mayer, 2014; Wentzel and Miele, 2016; Ryan and Rigby, 2019). In the game-based learning context, perhaps the most popular one refers to games’ pleasurable appeal and their ability to create enjoyable, interesting, and/or intrinsically motivating experiences. These experiences may result in learners trying harder and spending more time playing and, thereby, learning (Habgood and Ainsworth, 2011; Mayer, 2014).

Spending more time appears to be particularly relevant when repeated practice is needed to achieve the learning objective (Mayer, 2014), such as for automatizing grammar rules. Repeated practice *per se* can be boring, so one can speculate that, with a game, learners can learn more compared to a non-game intervention; this simply because they voluntarily engage in the game-afforded practice longer. Many language (Tsai and Tsai, 2018) and math (Tokac et al., 2019) learning games offer such repeated practice.

Most game-based learning studies have been conducted in labs or in classrooms with fixed or limited time-on-task (see e.g., Abdul Jabbar and Felicia, 2015; Boyle et al., 2016; but see also e.g., All et al., 2021, Study 2; Sýkora et al., 2021). Thus, the actual effect of game-derived enjoyment, or similar affective-motivational construct, on time-on-task was examined insufficiently. For example, a series of lab studies suggested that better-looking artistic design for a children’s educational game increased preference for interfacing with the better-looking game version, but not learning outcomes when the time-on-task was fixed (Javora et al., 2019, 2021a,b). The studies were inconclusive though with regards to the following question: what would happen had the children been allowed to learn from the game as long as they would have liked?

Contrary to popular beliefs, contemporary learning theories (Sweller et al., 2011; Plass and Kaplan, 2016; Mayer, 2021) imply that longer time-on-task with an educational game does not necessarily guarantee improved learning. Why? Educational games include playing and learning content. Learning from a game may be distractive compared to learning from more traditional materials such as animations or simulations. This is because the playing content competes with the learning content (as demonstrated, e.g., by Adams et al., 2012; Schrader and Bastiaens, 2012) for limited cognitive resources. Even if learners spent more time with an educational game than they did with non-game material, they may not have necessarily processed a higher amount of educational information during that time.

It is therefore useful to integrate the learning and the playing parts such that the distractive effect is minimized. One workable approach to such seamless integration stems from the idea to deliver “learning material through the parts of the game that are the most fun to play” (Habgood and Ainsworth, 2011, p. 173). This method has been called *intrinsic integration* in order to contrast it with approaches that use games as an extrinsic incentive or a “seductive” embellishment (see also Rey, 2012; Mayer, 2014). Worth noting, another alleged advantage of intrinsic integration is that it harnesses game-derived intrinsic motivation directly for the sake of learning (rather than extrinsic motivation). Habgood and Ainsworth (2011) in their seminar classroom study indeed demonstrated that a specific child game for promoting math practice, with intrinsic integration, notably prolonged time-on-task, and, at the same time, it enhanced learning outcomes (see also Bragg, 2012). However, this does not automatically imply that educational games with intrinsic integration are always better than comparable non-game materials, nor that intrinsic integration is necessarily distraction-free.

In this study, we examine whether a child educational game with intrinsic integration (inspired by the popular Moorhuhn^1^ shooting mechanic) for practicing a specific Czech language grammar rule affects time-on-task and the processing of educational information compared to a simple quiz with the same learning content. The game can be viewed as a model of games built around repeated quizzing (or, more broadly, repeated practice). Expanding the classical canon of small-scale laboratory experiments, we did a large field experiment. During a six-week period, thousands of children visiting, in their leisure time, Czech TV’s website with child online games could pick, among several dozens of other attractive games, our grammar training intervention. Unbeknown to them, they were assigned either to the game or the quiz condition. In such an experiment, one could not collect learning outcome data or demographic variables directly. Instead, by means of learning analytics, we examined variables related to participants’ persistence (time-on-task) and performance (numbers of tasks solved and errors). Our results complement existing game-based learning literature by bringing in data “from the wild” concerning the effectiveness of intrinsic integration and by offering insights on its possible pitfalls.

## This Study

In order to examine the effects of intrinsic integration on practice in informal settings, we picked the topic of enumerated words: a specific grammar element of the Czech language. Enumerated words represent those words and their variations in which the Czech language uses the letter *y* and not the letter *i* after particular consonants [e.g., brz**y** (soon), z**i**nek (zinc)]. In these words, using the letters *i*/*y* is interchangeable in the pronunciation, thus learning to write enumerated words requires memorization. These words are taught at Czech primary schools starting from Grade 2. This topic is in Czech generally viewed as difficult and boring.

**FIGURE 1:**
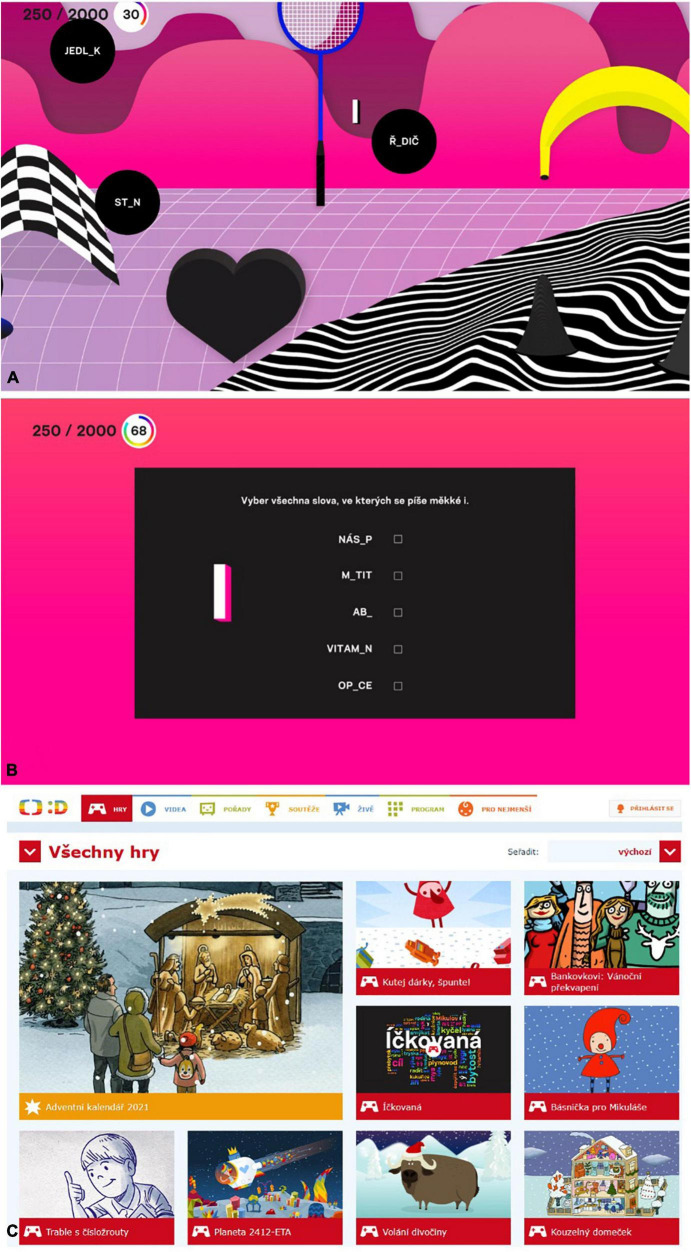
Demonstration of **(A)** the gameplay in the experimental intervention, **(B)** the control intervention and **(C)** the *ČT Déčko* website with the same banner for the *Íčkovaná* experimental and the control interventions (placed in the middle). [Courtesy of Czech Television (c)].

For the sake of this study, we developed two interventions: both called *Íčkovaná*. The experimental intervention is a game with graphical elements, a game environment and ambient background music and sound effects. Therein, participants shoot the chosen letter *i* or *y* on the words with an intentionally omitted vowel in the place where the letter *i*/*y* belongs in their pronunciations (Figure 1A). They shoot by clicking on the bubble with the word. Participants in the control group experienced a visually simple quiz with identical sound/music design in which they just clicked on the same words as in the game, still with omitted vowels, where they filled in the letter *i*/*y* (Figure 1B). The quiz presents five words: the number roughly equivalent to the average number of visible word-bubbles in the game. Both the quiz and the game have six levels with increasing difficulty. Each level can be played several times: we call passage through one level a *round* (no matter whether the player reached the required score or not).

### Research Questions

Based on the reasoning in the Introduction, we formed the following research questions:

Q1: Does the game “catch” player interest more than the quiz? That is, do more children complete at least one round in the game?

Q2: Does the game “hold” player interest more than the quiz? That is, (A) Do children play more game rounds in the game? (B) Do more children complete all six levels of the game? (C) Do more players return to the game/quiz, i.e., for more than one session?

Q3: Does the game distract attention away from the learning content? That is, (A) Do children complete fewer words in the game than they do in the quiz (i.e., by hitting/click on them)? (B) Do children make more grammatical errors in the game?

## Methods

### Participants and Design

Our participants were visitors to the official webpage of the public, mainstream television broadcaster Czech Television and its Déčko channel which has programming focused on younger audiences.^2^ This is a public, uncommercial website with a good reputation among parents. Roughly 20–30 thousand children visit this website daily; typically during their leisure time. We have collected anonymous data from those visitors that chose to use the *Íčkovaná* application. They could choose the app from a selection of more than 200 video games available on the site (Figure 1C) (*N* = 11,949 children, who clicked on the application). The app was featured prominently on the site to attract players (the banner was the same for both conditions so as not to disclose elements of the intervention; it only hinted that the app is about enumerated words). Due to the website’s anonymous nature, we could not collect any demographic information. However, given the profile of the site itself, its focus, Czech TV’s analytics and the intervention’s topic, we can assume the study participants were younger school-age children (age ∼8–10).

After clicking on the banner, children were randomly assigned to interface either with the quiz (*n* = 5867, 49.1%) or the game (*n* = 6082, 50.9%). When they returned for the second time, they were assigned the same version (through their browsers’ cache and stored cookies). The game was designed to be played primarily on computers.

### Interventions

#### Experimental Intervention

We created the *Íčkovaná* game as our experimental intervention specifically for the purpose of this study. It is a single-player, web-based game used to practice enumerated words. At the beginning of each of the six levels, players first choose whether they want to shoot the letter *i* or *y* at the words that lack this vowel in the given level (see Supplementary Table 1 for the list of words). Next, they engage in a single round of play for that level. Time allotment for one round is always 80 s (unless the player quits the round prematurely). The task is to locate words in the game environment and shoot the chosen letter at the words to which the selected letter belongs (see Figure 1A). The game includes horizontal parallax scrolling, where the farthest graphical layer is about three full screens wide. This sideways scrolling starts whenever the player’s cursor approaches the left or the right edge of the screen. This allows them to discover more floating words (see Supplementary Materials). Players usually have about 5 easy-to-reach words available on average.

After they appear in the lower half of the screen, each word slowly floats, for 10–14 s, toward the top of the screen where they disappear and cannot be shot at anymore. The score increases by 250 points for each correct shot and decreases by 250 points for each incorrect shot (i.e., the wrong letter). When the shot misses a word, the score is unaffected. In each level, players have to reach a minimum score in order to advance to the next level. In each consecutive level, the minimum score increases (see Supplementary Table 2) and the words are longer on average. The exact timing, parameters of the scoring system, and the overall difficulty was established during pilot testing, which also included qualitative research with children (*n* = 5). The game corresponds in complexity and visual esthetics to other games on the website^3^.

#### Control Intervention

A simple quiz on practicing enumerated words was used in the control condition. It shared several features of the game. It had six 80-second-long levels with the same scoring mechanism and pool of words for each level. At the beginning of each level, participants decided what letter (*i* vs. *y*) they wanted to fill into the blanks in the words at the given level. In each level, they were showed five words and were meant to click on those with the correct usage of *i*/*y*. Similar to the game, when players did not click on a word after 10 to 14 s, the word was replaced with a new one.

Participants experiencing the quiz did not use the shooting mechanic (the words did not move) and they did not look for the words in the game environment. The words were all listed in the middle of the screen (see Figure 1B). The quiz did not use game-like visuals, but rather minimalistic graphics. However, it shared the game’s audio/music design.

### Measures

We have collected our data exclusively using game telemetry. The nature of the collected data allowed us to analyze the actions of individual participants including their later returns to the game. At the same time, we were able to compare the actions of the intervention and the control group.

For the sake of this study, the following variables were inferred from raw data; for both interventions:

• maximum level that players finished at least once (i.e., by reaching the time limit, either successfully or unsuccessfully);
• maximum level that players finished successfully (by achieving the required score);
• number of rounds a player completed – successfully vs. unsuccessfully (the player could play a level more than once and could, but might not, achieve the required score);
• number of player sessions, i.e., how many times a player returned to the game, including the very first session (game vs. quiz);
• chosen letter *i*/*y* for each level;
• number of answer attempts per round (i.e., shots – game; clicks – quiz);
  ৹ number of correct answers (i.e., hits/clicks on words missing the letter *i*/*y* chosen at the beginning of this level);
  ৹ number of incorrect answers (i.e., hits/clicks on words missing the *other* letter *i*/*y*; that is, the letter other than the one chosen at the beginning of this level);
  ৹ number of misses (i.e., shots outside words – only in the game).

Full characteristics of raw data are included in Supplementary Table 3.

### Procedure

We collected data between August 26, 2021 and October 4, 2021. During this period, the *Íčkovaná* banner featured on the Déčko channel website (see Figure 1C). After clicking on the banner for the first time and subsequent assignment to the condition, we started collecting data. We saved all data after participants fully completed each level.

When a participant closed the page and later returned, they were assigned the same type of intervention with the same ID. Therefore, participants did not know about the existence of the second intervention.

### Data Analysis

Data were analyzed in software R (R Core Team, 2021). We primarily used *t*-tests to examine between-group differences. However, given the large skewness of the data, we also tested the differences using a non-parametric Mann–Whitney test and obtained similar results. For estimating effect sizes, we used Cohen’s *d* (for differences between means) and Cohen’s *h* (for difference between proportions): both use the same classification into small (∼0.2), medium (∼0.5), and large (∼0.8) effect sizes. When analyzing the game patterns, we removed an outlier with over 156 games played. We suspected this “player” to actually be a school with multiple students playing on the same computer.

### Ethics

The experiment was conducted as part of a larger project approved by the Ethics Committee of the Institute of Psychology of the Czech Academy of Sciences. No personal data was collected. The experiment used only fully anonymized telemetry data.

## Results

**FIGURE 2:**
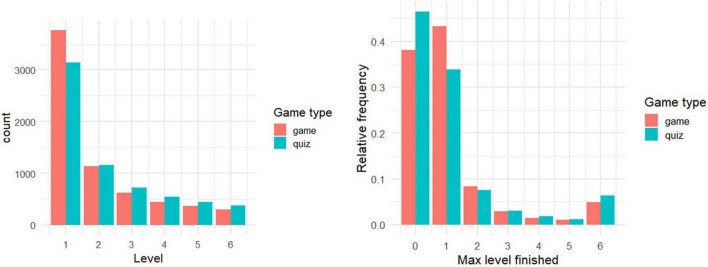
(Left) Number of players who completed either successfully or unsuccessfully *x^th^* level. I.e., all players in level *x* + 1 are also included in level *x*: the difference between levels *x* + 1 and *x* is the dropout rate between those two levels. Apparently, starting from level 2, more participants played the quiz. (Right) Relative percentage of players’ maximum level reached (out of players from each condition). That is, these players played the respective level at least once but might not necessarily have passed it successfully. The higher peak at level 6 corresponds to players who were determined to finish the game. Value 0 denotes players who did not finish even a single round.

Many players (*n* = 5045, 42.2%) stopped playing before finishing the first round (Figure 2); supporting the idea that the topic is boring. Participants picked *i*/*y* almost evenly (49.0% chose *y* across all plays), so we did not analyze this variable further.

### Q1: Does the Game “Catch” Player Interest More Than the Quiz? That Is, Do More Children Complete at Least One Level in the Game?

Game players were more likely to finish the first game round, be it successfully or unsuccessfully (61.8 vs. 53.6% out of all players in the respective condition). This difference in dropouts was statistically significant, although small [χ^2^(1) = 83.33; *p* < 0.001; Cohen’s *h* = 0.17, 95% CI (0.13, 0.20)]. We conclude that the game “catches” player interest more than the quiz as concerns finishing the first level.

### Q2: Does the Game “Hold” Player Interest More Than the Quiz? That Is, (A) Do Children Play More Game Rounds in the Game? (B) Do More Children Complete All Six Levels of the Game? (C) Do More Players Return to the Game/Quiz, i.e., for More Than One Session?

The number of players gradually decreased in subsequent levels (Figure 2). For further comparison, we focused only on players who finished the first level (either successfully or unsuccessfully). (A) These players, in game vs. quiz, did not differ in the total number of rounds played (Table 1, Line 1), so altogether these game and quiz players were engaged by the intervention for roughly the same amount of time. (B) Apparently, for some players, reaching the final level was important (Figure 2-right). Although quiz players were more likely to drop out during the first round, we see a comparably larger dropout rate between the first and the second level in the game condition (Figure 2-left). Moreover, quiz players were more likely to play until the final level compared to game players [6.0 vs. 4.3% of all participants assigned to the respective condition; χ^2^(1) = 17.15; *p* < 0.001, Cohen’s *h* = 0.08, 95% CI (0.04, 0.11); 11.2 vs. 7.0% of those who completed at least one round]. (C) Game sessions were significantly shorter than quiz sessions (Table 1, Line 2), so game players were engaged in more game sessions (i.e., returns to the game; Table 1, Line 3), but the differences were negligible. We conclude that there is not much support for the idea that the game holds players’ attention more than the quiz.

**TABLE 1:** Selected game/quiz parameters and their comparison for players who completed at least one round. We report *t*- and *p*- values from between-subject *t*-tests.

variable name	Game *M* (*SD*)	Quiz *M* (*SD*)	*t*	*p*	*d*	95% CI
1. Number of rounds a player completed (successful + unsuccessful)	2.78 (3.64)	2.70 (3.28)	0.95	0.343	0.02	(−0.02, 0.07)
2. Number of rounds played in one session	2.13 (2.10)	2.26 (2.15)	−2.44	0.015	−0.06	(−0.11, −0.01)
3. Number of player sessions	1.24 (0.81)	1.15 (0.54)	5.46	<0.001	0.13	(0.08, 0.18)
4. Maximum level that player finished (successful + unsuccessful)	1.76 (1.48)	2.03 (1.71)	−6.99	<0.001	−0.17	(−0.22, −0.12)
5. Maximum level that player finished successfully	1.16 (1.71)	1.62 (1.92)	−10.47	<0.001	−0.26	(−0.30, −0.21)
6. Number of answers per player (i.e., correct + incorrect)	52.39 (80.88)	60.06 (77.22)	−4.03	<0.001	−0.1	(−0.14, −0.05)
7. Number of correct answers per player	36.34 (53.18)	46.95 (65.38)	4.45	<0.001	0.1	(0.06, 0.15)
8. Number of incorrect answers per player	16.05 (33.60)	13.12 (20.42)	−7.3	<0.001	−0.18	(−0.23, −0.13)

### Q3: Does the Game Distract Attention Away From the Learning Content? That is, (A) Do Children Complete Fewer Words in the Game Than in the Quiz (i.e., By Hitting/Clicking on Them)? (B) Do Children Make More Grammatical Errors in the Game?

**FIGURE 3:**
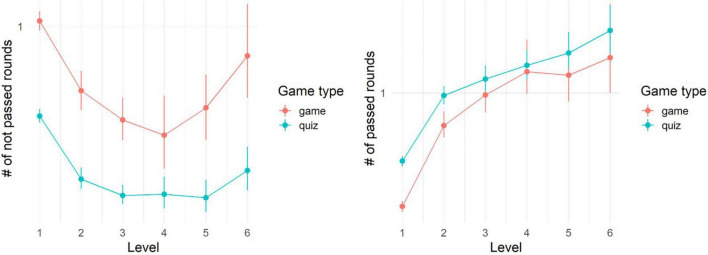
Mean number of not passed rounds per player and level (left) and passed rounds per player and level (right). Note that a player can return to the level and play it several times. Error bars indicate bootstrap-based 95%-confidence intervals.

After removing players who did not complete even one round, (A) the remaining players in the quiz successfully finished significantly more levels (Table 1, Line 5), answered significantly more words total (Line 6) and had (B) more correct answers (Line 7) and less wrong answers (Line 8) compared to the players in the game (see also Figure 3). Players in the game took an additional 9 shots (median; 25th and 75th percentiles: 3 and 30), which missed any presented word. We conclude that the game indeed is comparably more distractive.

## Discussion

The effectiveness of leisure time learning games has rarely been examined in real-world conditions. Here, we showed that when such a game/quiz for practicing specific grammar rules is available on a public children’s website featuring dozens of attractive online games, a notable portion of the site visitors voluntarily picks it and interfaces with it for at least a few minutes. During this period, they practice the grammar rules several dozen times. However, an almost equal number of children picks the game/quiz but then leaves it during the first level (i.e., during 80 s) without much of a practice. Compared to the quiz, the game successfully “catches” player interest: more of them stick with it for at least one round. However, the game is more distractive than the quiz: children answer fewer words in it and make more errors. Finally, the game does not “hold” player attention more so than the quiz: after the first level, more players stay with the quiz rather than the game.

The fact that children answered fewer words in the game and erred more is hardly surprising from the perspective of cognitive learning theories (e.g., Sweller et al., 2011; Plass and Kaplan, 2016; Mayer, 2021). Any learner, and children especially (Gathercole et al., 2004), have limited cognitive resources. While playing the game, they have to split these resources between learning and playing – this applies even to games with integrated mechanics. In our case, the children had to orient themselves in the game environment, locate targets, and then aim. None of this was necessary in the quiz. The game’s visual design and the mechanics distracted attention of children away from the learning task. In fact, even in the seminal study by Habgood and Ainsworth (2011), children were more accurate when solving mathematical tasks outside the game rather than within it; an often overlooked result of that study.

What is perhaps more surprising is the fact that the game, in terms of the four-phase interest development model (Hidi and Renninger, 2006), triggered (“catch”) situational interest but did not maintain it (“hold”). So, our players did not “try harder” in the game. This finding not only runs contrary to popular belief, but also to Habgood and Ainsworth’s findings and to some general multimedia learning research suggesting that better-looking visuals help maintain, rather than just trigger, interest (Endres et al., 2020). Additional research is needed to examine the reasons behind these contradictory results. We offer several tentative explanations.

First, the difference could be caused by the fact our intervention was not used as an assigned activity in a school or lab, but rather as a leisure time activity. Thus, children could freely pick from any of dozens of other games.

Second, there is emerging notion among multimedia learning researchers that design elements used to trigger interest should be unobtrusive (see e.g., Brom et al., 2018; Wong and Adesope, 2020). Game mechanics, even the integrated ones, might not always be as unobtrusive as one would wish; thus, thwarting interest and triggering frustration (e.g., “Gosh, I cannot find another bubble with the word to shoot at…”). We do not think this is the sole issue in the present case, because we have piloted the game and the mechanics used by Habgood and Ainsworth were not much different from ours. Still, this issue could have a contributing role.

Third, children might like the quizzing mechanics. Quizzing (or more generally, repeated testing) is an instructionally effective technique (Fiorella and Mayer, 2015; Wang and Tahir, 2020; Jičínská et al., 2021; Yang et al., 2021). However, Habgood and Ainsworth used, in their control condition, a non-learning combat game *interrupted* by a quiz between levels. This interrupted-game-quiz format may be more problematic compared to the mere quiz we or others used.

Finally, we can speculate that children who interfaced with the game/quiz longer were primarily driven by a need for achievement or topic interest (i.e., they wanted to see the “game over” screen or practice grammar rules) rather than by shooting/quizzing mechanics-derived interest. The latter idea is supported by a (weak) relationship between performance and persistence in our data (see section Supplementary Correlation Analysis). Plus, there is supporting evidence that topic-derived interest is more important than game-derived interest in game-based learning contexts (Brom et al., 2019). Future research can shed more light on these points.

### Limitations and Future Directions

First, we could not collect data on demographics, learning outcomes or perceived difficulty. This stems from the study design: we traded detailed information about each participant for a large sample and bird’s-eye view of field performance. Consequently, we could not examine underlying factors influencing children’s behavior when interfacing with the application (e.g., girls could have a different dropout in one condition then boys).

Second, for technical reasons, we could not log data on the children before they completed the first round of play, so we do not have information about what these participants did before they dropped out.

Third, some children were probably able to figure out that we had two intervention versions (e.g., when playing on two different computers) or siblings might use the same computer (i.e., be presented in logs as one participant). This could create some noise in the data, but we do not think this is a notable issue given the large sample size.

Finally, we examined one specific game-quiz couple with specific visuals and difficulty. It is not clear to what extent we could generalize our findings. In this study, we intentionally used as simple game as possible in order to make the interventions comparable. Additional between-intervention differences would create confounders, complicating interpretation of the findings. We now have information about our “basic” game version and we can compare it with game variants having more complex mechanics or different visual designs. This is our next step. Alternatively, one can conduct smaller-scale experiments, in which demographics can be collected in order to examine moderating factors. All in all, we think the study’s limitations present grounds for future research.

## Conclusion

The key take-home message from this study is that integrated mechanics in learning games are not a panacea. In the field, when competing with other activities, games with these mechanics may have the power to “catch” interest, but not necessarily to “hold” it. Plus, these games, like any other games, may have distractive elements. Persistence in playing them may not necessarily be driven by game mechanics. Altogether, these integrated mechanics, despite being useful starting points in designing new interventions, may also have some limitations.

## Data Availability Statement

The raw data supporting the conclusions of this article will be made available by the authors, without undue reservation.

## Ethics Statement

Ethical review and approval was not required for the study on human participants in accordance with the local legislation and institutional requirements. Written informed consent from the participants’ legal guardian/next of kin was not required to participate in this study in accordance with the national legislation and the institutional requirements. Additional information can be found in section “Ethics” above.

## Author Contributions

CB, KV, and LK designed the intervention. KV organized running the study during the experimental period. LK organized the data collection and wrote the first draft of section “This Study” and the supplement. FD and JL analyzed the data and wrote the first draft of section “Methods”. CB supervised the whole study and wrote the manuscript. All authors helped design the study and commented on the manuscript and interpretation of the findings.

## Conflict of Interest

KV declares a potential conflict of interest because she is employed by Czech TV, which is a public institution engaged (also) in development of educational games. The remaining authors declare that the research was conducted in the absence of any commercial or financial relationships that could be construed as a potential conflict of interest.

## Publisher’s Note

All claims expressed in this article are solely those of the authors and do not necessarily represent those of their affiliated organizations, or those of the publisher, the editors and the reviewers. Any product that may be evaluated in this article, or claim that may be made by its manufacturer, is not guaranteed or endorsed by the publisher.

## Footnotes

1 https://en.wikipedia.org/wiki/Moorhuhn (cited 2021-12-31).

2 https://decko.ceskatelevize.cz (in Czech, cited: 2021-12-31).

3 See the following link for video demonstration of the game and the quiz: https://www.frontiersin.org/articles/10.3389/fpsyg.2022.856623/full#supplementary-material.

## References

[B1] Abdul Jabbar A. I. Felicia P. (2015). Gameplay engagement and learning in game-based learning: A systematic review. *Rev. Educ. Res.* 85 740–779. 10.1093/geront/gnaa047 32530026 PMC8437506

[B2] Adams D. M. Mayer R. E. MacNamara A. Koenig A. Wainess R. (2012). Narrative games for learning: Testing the discovery and narrative hypotheses. *J. Educ. Psychol.* 104 235–249. 10.1037/a0025595

[B3] All A. Castellar E. N. P. Van Looy J. (2021). Digital Game-Based Learning effectiveness assessment: Reflections on study design. *Comput. Educ.* 167:104160. 10.1016/j.compedu.2021.104160

[B4] Boyle E. A. Hainey T. Connolly T. M. Gray G. Earp J. Ott M. (2016). An update to the systematic literature review of empirical evidence of the impacts and outcomes of computer games and serious games. *Comput. Educ.* 94 178–192. 10.1016/j.compedu.2015.11.003

[B5] Bragg L. A. (2012). The effect of mathematical games on on-task behaviours in the primary classroom. *Mathemat. Educ. Res. J.* 24 385–401. 10.1007/s13394-012-0045-4

[B6] Brom C. Dobrovolný V. Děchtěrenko F. Stárková T. Bromová E. (2019). It’s Better to Enjoy Learning than Playing: Motivational Effects of an Educational Live Action Role-playing Game. *Front. Learn. Res.* 7 64–90. 10.14786/flr.v7i3.459

[B7] Brom C. Starkova T. D’Mello S. K. (2018). How effective is emotional design? A meta-analysis on facial anthropomorphisms and pleasant colors during multimedia learning. *Educ. Res. Rev.* 25 100–119. 10.1016/j.edurev.2018.09.004

[B8] Cordova D. I. Lepper M. R. (1996). Intrinsic motivation and the process of learning: Beneficial effects of contextualization, personalization, and choice. *J. Educ. Psychol.* 88 715–730. 10.1037/0022-0663.88.4.715

[B9] R Core Team. (2021). *R: A Language and Environment for Statistical Computing.* Vienna, Austria: R Foundation for Statistical Computing.

[B10] Endres T. Weyreter S. Renkl A. Eitel A. (2020). When and why does emotional design foster learning? Evidence for situational interest as a mediator of increased persistence. *J. Comput. Assis. Learn.* 36 514–525. 10.1111/jcal.12418

[B11] Fiorella L. Mayer R. E. (2015). *Learning as a Generative Activity.* Cambridge: Cambridge University Press.

[B12] Garris R. Ahlers R. Driskell J. E. (2002). Games, motivation, and learning: A research and practice model. *Simul. Gam.* 33 441–467. 10.1177/1046878102238607

[B13] Gathercole S. E. Pickering S. J. Ambridge B. Wearing H. (2004). The structure of working memory from 4 to 15 years of age. *Dev. Psychol.* 40 177–190. 10.1037/0012-1649.40.2.177 14979759

[B14] Habgood M. J. Ainsworth S. E. (2011). Motivating children to learn effectively: Exploring the value of intrinsic integration in educational games. *J. Learn. Sci.* 20 169–206. 10.1080/10508406.2010.508029

[B15] Hidi S. Renninger K. A. (2006). The four-phase model of interest development. *Educ. Psychol.* 41 111–127. 10.1207/s15326985ep4102_4

[B16] Javora O. Děchtěrenko F. Tetourová T. Volná K. Brom C. (2021a). Customization in educational computer games and its effect on learning: Experimental study with primary school children. *J. Comput. Assist. Learn.* 37 1370–1382. 10.1111/jcal.12576

[B17] Javora O. Hannemann T. Volná K. Děchtěrenko F. Tetourová T. Stárková T. (2021b). Is contextual animation needed in multimedia learning games for children? An eye tracker study. *J. Comput. Assist. Learn.* 37 305–318. 10.1111/jcal.12489

[B18] Javora O. Hannemann T. Stárková T. Volná K. Brom C. (2019). Children like it more but don’t learn more: Effects of aesthetic visual design in educational games. *Brit. J. Educ. Technol.* 50 1942–1960. 10.1111/bjet.12701

[B19] Jičínská L. Sedláčková P. Kolek L. Tetourová T. Volná K. Lukavský J. (2021). Extrinsically integrated instructional quizzes in learning games: an educational disaster or not? *Front. Psychol.* 12:678380. 10.3389/fpsyg.2021.678380 34489794 PMC8417244

[B20] Malone T. W. (1981). Toward a theory of intrinsically motivating instruction. *Cogn. Sci.* 5 333–369. 10.1207/s15516709cog0504_2

[B21] Mayer R. E. (2014). *Computer games for learning: An evidence-based approach.* Cambridge: MIT Press.

[B22] Mayer R. E. (2021). *Multimedia Learning (3nd ed.).* Cambrige: Cambridge University Press.

[B23] Plass J. L. Kaplan U. (2016). “Emotional design in digital media for learning,”, in *Emotions, technology, design, and learning*, eds Tettegah S. Y. Gartmeier M. Elsevier: Academic Press, 131–161. 10.1016/b978-0-12-801856-9.00007-4

[B24] Rey G. D. (2012). A review of research and a meta-analysis of the seductive detail effect. *Educ. Res. Rev.* 7 216–237. 10.1016/j.edurev.2012.05.003

[B25] Ryan R. M. Rigby C. S. (2019). “Motivational foundations of game-based learning,”, in *Handbook of game-based learning*, eds Plass J. L. Mayer R. E. Homer B. D. (Cambrige: The MIT Press), 153–176.

[B26] Schrader C. Bastiaens T. J. (2012). The influence of virtual presence: Effects on experienced cognitive load and learning outcomes in educational computer games. *Comput. Hum. Behav.* 28 648–658. 10.1016/j.chb.2011.11.011

[B27] Sweller J. Ayres P. Kalyuga S. (2011). *Cognitive Load Theory.* New York: Springer.

[B28] Sýkora T. Stárková T. Brom C. (2021). Can narrative cutscenes improve home learning from a math game? An experimental study with children. *Brit. J. Educ. Technol.* 52 42–56. 10.1111/bjet.12939

[B29] Tokac U. Novak E. Thompson C. G. (2019). Effects of game-based learning on students’ mathematics achievement: A meta-analysis. *J. Comput. Assist. Learn.* 35 407–420. 10.1111/jcal.12347

[B30] Tsai Y. L. Tsai C. C. (2018). Digital game-based second-language vocabulary learning and conditions of research designs: A meta-analysis study. *Comput. Educ.* 125 345–357. 10.1016/j.compedu.2018.06.020

[B31] Wang A. I. Tahir R. (2020). The effect of using Kahoot! for learning–A literature review. *Comput. Educ.* 149:103818. 10.1016/j.compedu.2020.103818

[B32] Wentzel K. R. Miele D. (2016). *Handbook of Motivation at School (2*^nd^* ed.).* Oxfordshire: Routledge.

[B33] Wong R. M. Adesope O. O. (2020). Meta-analysis of emotional designs in multimedia Learning: A Replication and Extension Study. *Educ. Psychol. Rev.* 33 357–385. 10.1007/s10648-020-09545-x

[B34] Yang C. Luo L. Vadillo M. A. Yu R. Shanks D. R. (2021). Testing (quizzing) boosts classroom learning: A systematic and meta-analytic review. *Psychol. Bull.* 147 399–435. 10.1037/bul0000309 33683913

