# Supplementary material for: To Quiz or to Shoot When Practicing Grammar? Catching and Holding the Interest of Child Learners: A Field Study
Source: Front Psychol. 2022 Apr 14;13:856623. doi: 10.3389/fpsyg.2022.856623 (PMC9049184; doi:10.3389/fpsyg.2022.856623)
Supplement: Supplementary file 1 [file Data_Sheet_1.PDF]

Supplementary Material

Table S1. List of words in each level. This character "_" represents in each word the part of it in which the correct letter had to be added. The correct letter i or y to complete the word immediately follows it.

	Words missing *i* in the first level	Words missing *y* in the first level	Words missing *i* in the second level	Words missing *y* in the second level	Words missing *y* in the third level	Words missing *y* in the third level	Words missing *y* in the fourth level	Words missing *y* in the fourth level	Words missing *y* in the fifth level	Words missing *y* in the fifth level	Words missing *y* in the sixth level	Words missing *y* in the sixth level
3 letters	c_íl	b_ýk	j_ít	t_yč	s_íť	k_ýl						
	z_ip	s_yn	j_iž	m_yš	p_ít	d_ým						
	as_i	k_yj	f_ík	p_ýr								
	š_ít	ab_y	ž_ít	s_ýr								
	l_ít	h_ýl										
	n_ic	r_ys										
4 letters	b_ída	dud_y	d_ítě	vžd_y	ž_íla	t_ygr	št_ír	m_ysl				
	bd_ít	l_ýko	mez_i	brz_y	zel_í	l_yže	ct_ít	om_yl				
	m_ilá	r_ýha	ryz_í	noh_y	_igor	pl_yn	ml_ít	_yzop				
	č_íst	hm_yz	d_íra	sm_yk	t_íha	pl_yš	j_íst	d_ýka				
	ciz_í	m_ýto	s_íra	l_ysý	m_imo	m_ycí	ř_íct	r_yba				
	s_ivá	L_ysá nad Labem	_ivan	d_ýně								
	st_ín	p_ysk	č_ilý	zv_yk								
	J_iří	r_ýma	dř_ít	vl_ys								
	l_ípa	s_ytý	v_ina	tud_y								
	As_ie	ml_ýn	dět_i	r_ýže								
5 letters	cen_it	b_ytná	n_ikdo	s_ykot	has_it	p_ytel	kos_it	v_ýška	l_iška	v_ýběr		
	ř_idič	k_yčel	ř_ízek	sm_ysl	ruš_it	kr_ysa	poc_it	běžk_y	J_itka	k_ypřit		
	L_inda	nerv_y	zm_ije	v_ydra	nos_it	ťuh_ýk	sép_ie	m_ýdlo	Jul_ie	jaz_yk		
	op_ice	C_yril	t_isíc	s_yčet	_irena	b_ytná	š_iška	m_ýlit se	Mar_ie	kor_ýš		
	n_ikdy	r_ydlo	kaz_it	ror_ýs	jmel_í	r_ybíz	l_ilek	b_ytná	zaj_íc	k_ývat		
	rad_it	hezk_y	c_ivět	L_ýdie	M_ilan	d_ýmat	p_izza	nůžk_y	_iluze	m_yšák		
	n_ičit	v_yšší	Kol_ín	p_ýcha	tal_íř	s_ysel	dř_ina	v_ýkal	d_ivák	pěšk_y		
	l_išit se	b_ývat	pál_it	m_ýtus	t_icho	H_ynek	č_ížek	r_ybák	p_írko	V_ýtoň		
	sv_išť	p_yšně	dus_it	t_yčka	ryt_íř	v_ýlov	bav_it	pos_yp	hřm_ít	úm_ysl		
	Luc_ie	pov_yk	rub_ín	sum_ýš	lep_it	někd_y	div_it se	v_ykuk	velm_i	v_ýheň		
	V_ítek	vým_yk	zab_ít	l_ýtko	koš_ík	mot_ýl	kal_it	m_ýlka				
	Š_imon	t_ýden	c_ídit	Kam_ýk	kř_ída	t_ykev						
	zač_ít	p_yšný	D_iana	h_ýkat	Děč_ín	kdyb_y						
	kon_ík	nás_yp	Dav_id	t_yran	líb_it se	p_ýřit se						
	ud_ice	kd_ysi	Žof_ie	h_ymna	J_ičín	obr_ys						
	bal_it	zás_yp	c_ítit	m_ýval	naj_ít	s_ypká						
	F_ilip	s_ypek	č_íslo	m_yčka	š_ípek	d_ýmka						
	M_iloš	m_ýtit	_ivana	s_ýček								
	fot_it	v_ýron	s_irup	v_yžle								
	bud_ík	s_ynek	l_išaj	v_ývar								
	děl_it	brzk_ý										
	plš_ík	kur_ýr										
	buš_it	čt_yři										
	v_išeň	v_ýčep										
	v_idle	náv_yk										
	cht_ít	v_ykat										
6 letters	rod_ina	l_ysina	v_iolka	zlos_yn	baž_ina	pozb_ýt	pl_ížit se	v_ýroba	vid_ina	kost_ým	chyt_it	nezv_yk
	n_ičema	tr_yska	koup_it	nejv_ýš	pav_ián	m_ýtina	kuřec_í	p_ytlák	las_ice	v_ýsost	hověz_í	Ruz_yně
	l_imeta	sm_ýkat	odst_ín	k_ytara	Jud_ita	zv_ýšit	orl_ice	pl_yšák	brán_it	m_yslet	kamz_ík	ob_yčej
	krm_ivo	b_ytost	zloč_in	kor_yto	Kouř_im	sl_yšet	op_ičit se	kr_ypta	kř_ičet	hr_yzat	L_ibuše	v_ýloha
	jedl_ík	úb_ytek	vač_ice	b_ydlet	kř_ižák	krun_ýř	včel_ín	V_yškov	vel_ice	r_ybina	hlad_it	moh_yla
	cv_ičit	d_ýchat	N_ikola	kop_yto	Ct_ibor	kr_ysař	král_ík	ob_ydlí	svač_it	S_yslov	c_itron	chm_ýří
	básn_ík	žv_ýkat	peř_ina	trenk_y	loup_it	t_ymián	děj_iny	fen_ykl	mrač_it se	pom_yje	cedn_ík	r_ypouš
	brhl_ík	k_yslík	pušt_ík	pl_ytký	bl_ízko	jakob_y	hlás_it	zv_ykat	M_ilena	h_ýčkat	pěn_ice	Mat_yáš
			nutr_ie	r_ytmus	nářad_í	Jách_ym	kl_íště	skr_ytě	tanč_it	sm_yčec	m_íchat	Ot_ýlie
			nočn_ík	dějin_y	Měln_ík	bab_yka	ručn_ík	zb_ytek	slav_ík	holin_y	bl_ikat	čt_yřka
					rohl_ík	b_ývalý	norn_ík	m_ydlit	sl_imák	hr_yzec	V_iktor	ch_yták
					sv_ítit	kob_yla	Afr_ika	v_ýznam	p_ilník	s_ýkora	vavř_ín	plavk_y
					Mart_in	oz_ývat se	houšt_í	b_ystrá	araš_íd	sm_ýčit	krt_ina	Vol_yně
					c_ibule	ml_ynář	mal_ina	kuch_yň	hod_ina	Bos_yně	Mon_ika	dům_ysl
							J_ílové	v_ýskat	Hoř_ice	ch_ybět	Kam_ila	ob_ývat
									Př_imda	sm_yčka	delf_ín	cav_yky
									ol_iheň	v_ysoká	hř_íčka	plat_ýs
									ž_irafa	Zb_yšek	Dobř_íš	B_ydžov
											ang_ína	s_ytost
7 letters	obj_ímat	v_ýprava	druž_ice	nesm_ysl	otráv_it	velr_yba	d_ivadlo	průsm_yk	pevn_ina	v_yrážka	j_iřička	zam_ykat
	vitam_ín	čep_ýřit se	kobl_iha	sl_ynout	brous_it	bl_yštět se	vakc_ína	jaz_ýček	gum_ička	dob_ytek	bř_inkat	záclon_y
	otazn_ík	Přem_ysl	Jarom_ír	v_ýstava	l_ibeček	v_ynadat	chř_ipka	pl_ynule	barv_ivo	v_ýmluva	papr_ika	r_ýsovat
	M_ikulov	spl_ývat	chrán_it	třp_ytka	nov_inka	kob_ylka	Hodon_ín	us_ychat	gr_izzly	vzl_ykot	ledn_ice	pov_ýšit
			bubl_ina	v_yčůrat se	okamž_ik	v_yvářet	část_ice	kalhot_y	hv_ízdat	v_yhublý	poč_ítač	v_ykydat
			B_ílovec	v_ýborně	paž_itka	r_ybolov	mam_inka	zal_ykat se	tipl_ice	zab_ývat se	Anton_ín	S_ychrov
			nám_itka	naz_ývat	kon_ipas	kr_ystal	M_ikuláš	Hrab_yně	podiv_ín	pel_yněk	J_ihlava	bl_ýskat se
			konč_ina	skr_ývat	skř_ivan	Mat_ylda	ž_ihadlo	s_ynovec	Dal_ibor	nas_ytit	kresl_it	v_ýpomoc
			trén_ink	zlozv_yk	Hav_ířov	přep_ych	objev_it	r_ybenka	slan_ina	prům_ysl	klad_ivo	pol_ykat
			krab_ice	t_yčinka	chlub_it se	obv_ykle	vaj_íčko	Zb_yněk	kraj_ina	třp_ytit se	odč_ítat	v_ýrobek
							druž_ina	Zb_yslav	osm_ička	b_ytelný	deštn_ík	netop_ýr
							kul_íšek	Hor_ymír	kopř_iva	v_ymykat se	roz_inka	r_ychlík
							M_ichael	r_ychlík	nakaz_it	pl_ynout	sklen_ík	dupačk_y
							š_impanz	vzl_ykat	svač_ina	t_ykadlo	obv_init	v_ýhodný
											slep_ice	pl_ýtvat
											polož_it	hlem_ýžď
											drač_ice	náb_ytek
											Chrud_im	nas_ypat
											Bohum_ín	s_yreček
											Karv_iná	dm_ýchat
8 letters					kruž_ítko	V_yšehrad	brus_inka	V_ysočina	P_ilníkov	klop_ýtat	vi_dlička	pl_ynovod
					kapesn_ík	rozmar_ýn	M_iroslav	p_ýchavka	chud_inka	s_ychravo	kružn_ice	příb_ytek
					Kateř_ina	zas_ychat	ant_ilopa	neom_ylně	blud_ička	v_ypasený	m_íchačka	m_yslivec
					jept_iška	v_ýrostek	J_indřich	v_ychrtlý	pískom_il	sm_ýšlení	kukuř_ice	přeb_ytek
					marc_ipán	zv_yklost	lékoř_ice	vypl_ývat	plav_idlo	nadb_ytek	p_itomost	Valent_ýn
					blud_iště	živob_ytí	hvězd_ice	m_yšlenka	rosn_ička	přim_ykat	hrdl_ička	zb_ytečně
							Kop_idlno	um_yvadlo	budn_íček	Litom_yšl	řebř_íček	ob_yvatel
							Javorn_ík	kap_ybara	kost_ival	pov_ýšená	kap_itola	L_ysolaje
							zelen_ina	vyz_ývavý	žehl_ička	blahob_yt	sv_íčková	zlatob_ýl
									zmrzl_ina	zp_ytovat	zloč_inec	odem_ykat
									obratn_ík	m_yslivna	Veron_ika	pl_ynárna
									bláz_inec	vym_yslet	p_ísnička	b_ystřina
											j_itrocel	žv_ýkačka
											karaf_iát	b_ydliště
											p_ískomil	V_ysočany
