# Supplementary material for: To Quiz or to Shoot When Practicing Grammar? Catching and Holding the Interest of Child Learners: A Field Study
Source: Front Psychol. 2022 Apr 14;13:856623. doi: 10.3389/fpsyg.2022.856623 (PMC9049184; doi:10.3389/fpsyg.2022.856623)
Supplement: Supplementary file 2 [file Data_Sheet_2.pdf]

frontiers

Supplementary Material

Table S2. Minimal scores for each of the six levels.

Level number	Minimal score
1	2000
2	2250
3	2500
4	2750
5	3000
6	3250

Table S3. List of variables collected using game telemetry.

Name	Description	Values	When collected?*
user_id	Identification of the individual participant	string	beginning; level
game_id	Identification of the individual game session	string	beginning; level
log_time	Time log of the beginning of the game session	timestamp	beginning; level
game_type	Type of the intervention	1 = game<br>2 = quiz	beginning; level
correctNum	Number of correct hits on target	integer	level
escaped	Indicator whether players left the intervention using the game menu	boolean	level
chosen_y	Indicator whether participants chose to fill in the letter "y" or "i"	boolean	level
level_num	Level identification	range 1 to 6	level
passed	Indicator whether participants reached the minimum score	boolean	level
shotsNum	Total number of shots done on and off target	integer	level
wrongNum	Number of incorrect hits on target	integer	level
id	Identification of the individual word	integer	hit; word

word	Particular word	string	hit; word
correct	Identification whether the hit on target was correct or not	boolean	hit
is_y	Identification of whether the letter "y" or "i" was correct	boolean	hit
level_time	Time log of the particular hit	range 0 to 80 seconds	hit
pos_x	Position of the word on the x-axis in time of the hit	integer	hit
pos_y	Position of the word on the y-axis in time of the hit	integer	hit
pos_x	Position on the x-axis when the word appeared on the screen	integer	word
pos_y	Position on the y-axis when the word appeared on the screen	integer	word
spawn_level_time	Time log of when the word appeared in the level	range 0 to 80 seconds	word

**beginning = at the beginning of the intervention*
level = for each level individually
hit = for each hit on the target words
word = for each individual word in each level

Section Supplementary Correlational analysis.

We additionally asked whether or not players having higher scores in the first round stayed with the intervention longer.

After removing players who did not complete even one round, associations between performance in the first round played (number of correct and wrong responses) and the total number of rounds played were significant but small (wrong responses: Spearman ρ -0.13 for game and -0.23 for quiz; correct responses: 0.05 for game and 0.04 for quiz; all $p < 0.05$).

This means that players who were better during the first level stayed with the intervention slightly longer. One possible explanation is that those better at the beginning appreciated they can practice this specific grammar rule, improving their skills. However, there are also alternative explanations. For instance, it is possible that older participants better know the grammar rule and, at the same time, can focus on practicing the task longer (because of their age) – so the underlying cause could be age. Based on a correlational analysis from a field experiment, we cannot say much about underlying causes; future research collecting demographics would be needed.
